# A review on the structure and function of plant thaumatin-like proteins

**DOI:** 10.3389/fpls.2026.1797768

**Published:** 2026-04-23

**Authors:** Yuanyuan Liu, Jinzhu Li, Wei Wang, Lu Yang, Xinjie Zhang, Jie He, Yuan Wang, Feng Wei, Zhanshuai Li, Jun Peng

**Affiliations:** 1 Nanfan Research Institute, Chinese Academy of Agricultural Sciences, Sanya, China; 2 Institute of Cotton Research, Chinese Academy of Agricultural Sciences, Anyang, China; 3 Institute of Western Agriculture, Chinese Academy of Agricultural Sciences, Changji, China

**Keywords:** function, pathogenesis-related protein, stress resistance, structural characteristics, thaumatin-like protein

## Abstract

Thaumatin-like proteins (TLPs), a subclass of pathogenesis-related proteins (PR5) distinguished by the unique and stable “TLP-fold” structure, are ubiquitously found across plants, animals, and fungi. TLPs play a pivotal role as effector molecules in plant antimicrobial defense, while their structural flexibility and involvement in complex regulatory networks allow them to integrate multiple phytohormone signals, including salicylic acid, jasmonic acid, ethylene, and abscisic acid. This enables a coordinated response to both biotic and abiotic stresses, positioning TLPs as an “integrative hub” in plant stress adaptation. This review synthesizes the current literature on gene functional validation, providing a systematic overview of the structural characteristics, evolutionary features, and regulatory mechanisms of the TLP family. It emphasizes the multifunctionality and molecular mechanisms of TLPs in disease resistance, stress tolerance, growth regulation, and their role as allergens. Additionally, it discusses the central role of TLPs in integrating stress responses and their potential applications in crop resistance breeding, offering valuable insights for future research.

## 1 Introduction

As key components of the biosphere, plants are continuously subjected to a range of biotic and abiotic stresses from their environment (Atkinson and Urwin, 2012). To defend against these threats, plants have evolved sophisticated immune and adaptation systems. Within these, pathogenesis-related (PR) proteins play pivotal roles in rapidly responding to stress, executing direct defense, and facilitating signal transduction (Boller and Felix, 2009; Ahuja et al., 2012).

Among the 17 families of PR proteins, *Thaumatin-like* proteins (TLPs) within the pathogenesis-related protein 5 (PR5) family have garnered significant attention. TLPs are multifunctional proteins found in plants, animals, and fungi (Lr. et al., 1996; Shatters et al., 2006) characterized by evolutionary conservation and functional diversity. Initially isolated from the tropical rainforest plant *Thaumatococcus danielli* L (Wel and Loeve, 1972), TLPs attracted attention due to their intensely sweet taste. However, further investigation into their genomic characteristics, structure, and functions has revealed that they not only possess sweetening properties but also play critical roles in plant defense and growth regulation. TLPs actively respond to biotic stresses caused by microorganisms, pests, and other organisms, as well as abiotic stresses from environmental factors, exhibiting antifungal activity, osmotic regulation, allergenicity, and enzyme inhibition. Furthermore, TLPs can inhibit fungal enzymes such as xylanase, amylase, and trypsin, reduce spore viability, and disrupt fungal cell membranes. Notably, TLPs can sense and integrate multiple signals and are regulated by various phytohormones or stress factors such as SA, JA, ET, and ABA, allowing them to participate in multiple defense pathways, though they function primarily as downstream executors rather than as central signaling integrators.

In addition to exploring their structure and function, there is a growing emphasis on understanding the synthesis and regulatory mechanisms of TLPs within plants, as well as their practical applications. In recent years’ research, rapid advancements in structural biology, particularly X-ray crystallography, have enabled the determination of the three-dimensional structures of several plant TLPs, including thaumatin and monellin (Ogata et al., 1992; Batalia et al., 1996; Koiwa et al., 1999). These studies have clarified the composition of their structural domains, folding patterns, and key features related to their sweetening properties (Jesús-Pires et al., 2024). Functionally, differences in sweetness among various plant TLPs have been linked to specific amino acid sequences, with some proteins being hundreds of times sweeter than sucrose (Wintjens et al., 2011). Additionally, certain TLPs exhibit insect resistance and antimicrobial activities; for instance, some can trigger immune responses in insects, thereby displaying insecticidal effects (Qin et al., 2020). Research has also highlighted the role of TLPs in plant responses to environmental stresses, including drought and high temperatures, focusing on their expression regulation and potential functions under such conditions (Zhao et al., 2021).

This review focuses on the plant TLP gene family, aiming to elucidate their evolutionary origins and genomic distribution characteristics. We particularly emphasize the relationship between their highly conserved protein structure and functional diversity. In this review, we conceptualize TLPs as “integrative hubs” that integrate multiple stress signals and coordinate plant defense and development through three interconnected mechanisms: (1) Protein-Protein Interaction Networks: Engaging in physical interactions with diverse signaling proteins via their structural domains (particularly the TLP-PA domain), forming complex signaling networks; (2) Shared Signaling Nodes: Simultaneously participating in multiple phytohormone signaling pathways (SA, JA, ET, ABA), functioning as signaling intersection; (3) Transcriptional Co-regulation Networks: Their expression is coordinately regulated with other defense-related genes, responding to common upstream transcription factors. We thoroughly outline their roles and molecular mechanisms in growth regulation and environmental stress responses, systematically examining how these mechanisms enable TLPs to function as central integrators. Additionally, we discuss their potential applications in crop resistance breeding, plant protection, and the development of novel biopreparations. This review is intended as a resource for future research and offers a forward-looking perspective on emerging research directions.

## 2 Evolution and distribution of the TLP gene family

PR proteins are critical defense molecules induced in plants in response to pathogen infection, directly contributing to disease resistance mechanisms. The PR protein family is composed of 17 distinct classes, with TLPs representing the sole members of the PR-5 family, comprising 15-20% of all PR proteins. TLPs function as the “central functional component” of the PR protein family, seamlessly bridging basal defense with advanced immune responses.

**Figure 1:**
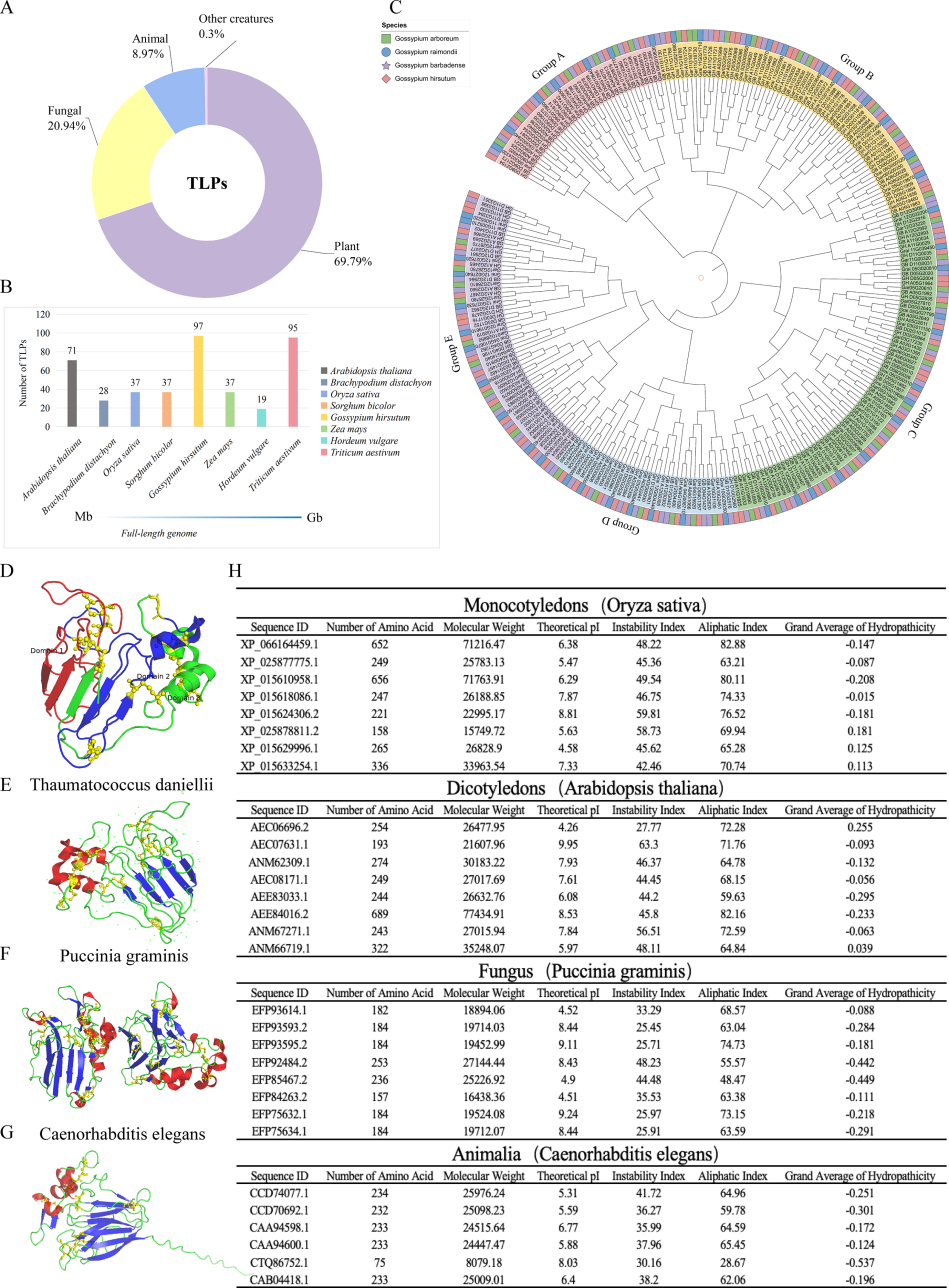
Evolution and distribution of the *TLP* gene family. **(A)** Estimation and statistical analysis reveal the ancient origin and wide distribution of TLPs across kingdoms. Annotation data from sequenced species in NCBI, UniProt, and Phytozome databases. **(B)** Gene copy number variation among species correlates with genome polyploidy and complexity. The x-axis represents genome length, and the y-axis represents the number of TLPs. **(C)** In Gossypium, TLPs cluster into distinct clades, showing lineage-specific expansion after polyploidization. Phylogenetic tree of *Gossypium arboreum*, *G. raimondii*, *G. hirsutum*, and *G. barbadense*, constructed using the neighbor-joining method with 1000 bootstrap replicates. Five different groups are highlighted with different background colors. Percentages next to branches represent the proportion of replicate trees where associated taxa clustered together in the bootstrap test (1000 replicates). Evolutionary distances were computed using the Poisson correction method and are in units of amino acid substitutions per site. Evolutionary analyses were conducted in MEGA (X) **(D)** The conserved ‘TLP-fold’ structure with eight disulfide bonds (yellow sticks) underlies its stability and function. Data from the PDB protein database, visualized with PyMOL, divided into three domains represented in red, green, and blue. Yellow stick-and-ball models represent disulfide bonds. **(E-G)** Structural comparison highlights conserved domains across plants, fungi, and animals. Representative structures: *Thaumatococcus daniellii* (PDB: 1THV) for plants, *Puccinia graminis* (PDB: 7P23) for fungi, and *Caenorhabditis elegans* (Uniprot prediction: thn-4) (Uniprot: https://www.uniprot.org/blast) for animals. α-helices are shown in red, β-sheets in blue, random coils in green, and disulfide bonds as yellow sticks. **(H)** Analysis of physicochemical properties of TLPs from dicots, monocots, animals, and fungi. It indicates adaptation to different ecological niches. The figure shows a partial list; see supplementary table for complete results.

The *TLP* gene family is an ancient and widely distributed multigene family found across plants, animals, and fungi, as illustrated in **Figure 1A**, suggesting a common ancestral gene. Genomic database searches (NCBI, UniProt, Phytozome) have identified TLPs in hundreds of sequenced species across plants, fungi, and animals, with particularly extensive expansion in plant genomes where copy numbers range from fewer than 20 in some species to nearly 100 in polyploid crops like wheat. The widespread distribution and high copy numbers in many genomes suggest TLPs represent one of the most abundant PR protein families in nature (de Jesús-Pires et al., 2020). The majority (approximately 70%) of TLPs are found in plants, highlighting their central role in plant stress resistance. Fungal TLPs represent a “double-edged sword,” with roughly 21% possessing both defensive and offensive functions (Hu et al., 2025). These proteins participate in cellular metabolism as defense molecules while also serving as virulence factors that aid infection. Animal TLPs constitute only 9%, possibly due to partial replacement by acquired immune systems.

Research on plant sweet proteins provided the foundation for understanding TLP functional diversity. In the 1960s-70s, Kurihara Kenzō isolated *miraculin* (Kurihara and Beidler, 1968) from *Synsepalum dulcificum*, which altered taste perception by converting sour to sweet. In the 1980s-90s, Ding Ming and Göran Hellekant isolated and characterized *brazzein* (Ming and Hellekant, 1994) from *Pentadiplandra brazzeana* B., determining its molecular weight and amino acid sequence. These early studies provided the first purified TLPs for structural analysis. Subsequently, the unexpected discovery of antifungal activity in tobacco PR5 family members revealed that TLPs possess functions beyond sweetness perception. To date, seven sweet proteins have been discovered in plants, including *thaumatin*, *mabinlin*, *brazzein*, *monellin*, *miraculin*, *pentadin*, and *curculin* (Masuda and Kitabatake, 2006; Wintjens et al., 2011). The number, structure, and function of *TLP* family members vary across species, likely reflecting their evolutionary history and ecological environment.

Early studies reported the presence of TLPs in basidiomycete fungi, such as *Irpex lacteus* (Fr.), *Lentinula edodes* (Berk.), and *Rhizoctonia solani* (J.G. Kühn) (Grenier and Asselin, 2000). Fungal TLPs typically exhibit β-1,3-glucanase activity, playing roles in cell wall degradation and spore dispersal of fruiting bodies (Sakamoto, 2006). Later studies identified 10 proteins containing thaumatin-like domains in the Rhizoctonia solani strain AG4-JY (Liu et al., 2024a).

TLPs in plants have been extensively studied, with over 180 plant species identified to harbor varying numbers of these proteins, spanning dicots, monocots, gymnosperms, bryophytes, and algae (de Jesús-Pires et al., 2020). Whole-genome analyses show that the *TLP* gene family has undergone significant expansion and differentiation across the plant kingdom. The number of TLPs correlates with plant genome ploidy and complexity. For instance, reference genomes of diploid crops such as barley, maize, rice (*Oryza sativa*), *Brachypodium distachyon*, and *Sorghum bicolor* contain 19, 37, 37, 28, and 37 *TLP* genes, respectively. In contrast, wheat (*Triticum aestivum*) harbors up to 95 *TLP* genes (Iqbal et al., 2020; Sharma et al., 2022; Zhao et al., 2024). This variation likely results from gene duplication events associated with polyploidization (**Figure 1B**). Additionally, dicots generally possess more *TLPs* than monocots, a trend potentially driven by natural selection favoring the retention of fewer duplicated genes in monocots during evolution. A total of 47, 49, 90 and 90 *TLP*s were respectively identified in the important economic crops of cotton (*Gossypium arboreum*, *G. raimondii*, *G. hirsutum*, and *G. barbadense*) (Cao et al., 2026). Phylogenetic analysis (**Figure 1C**) reveals that these *TLPs* can be grouped into five distinct clusters. Diploid cotton species exhibit a more even distribution of TLPs, whereas allotetraploid species show a more clustered evolutionary pattern, indicating significant specific differentiation. After allopolyploidization, these species inherited homologous genes from the A and D ancestors, with partial differentiation occurring over time.

TLPs were first discovered in animals in *Caenorhabditis elegans* (*Maupas*) (Kitajima and Sato, 1999) followed by reports in *Schistocerca gregaria* (Forsskål) (Brandazza et al., 2004), *Acyrthosiphon pisum*, *Tribolium castaneum* (Herbst) (Liu et al., 2010), and other insects across four orders: Coleoptera (e.g., *Diaprepes* and *Biphyllus*), Hemiptera, Hymenoptera (e.g., *Lysiphlebus*), and Orthoptera (e.g., *Schistocerca*) (Shatters et al., 2006). The widespread distribution and high diversity of TLPs in nature provide strong evidence of their irreplaceable biological functions and evolutionary significance. They represent a key survival advantage granted by natural selection, serving as strategic molecular tools for environmental adaptation in both plants and other eukaryotes.

## 3 Structural characteristics of the TLP gene family

The *TLP* gene family is classified as a multigene family (Zhao and Su, 2010). Despite their diversity, TLPs share a highly conserved “TLP-fold” with similar conserved motifs: G-x-[GF]-x-C-x-T-[GA]-d-C-x(1,2)-[GQ]-x(2,3)-C (Tachi et al., 2009) where standard IUPAC notation is used: G = glycine, x = any amino acid, [GF] = either glycine or phenylalanine, C = cysteine, T = threonine, [GA] = either glycine or alanine, d = aspartic acid (using single-letter code), x(1,2) = 1–2 any amino acids, [GQ] = either glycine or glutamine. The cysteine residues in this motif are particularly critical as they form disulfide bonds essential for structural stability. All TLPs contain the thaumatin domain (PF00314), which is highly conserved. For instance, in *Thaumatococcus daniellii* (**Figure 1D**), crystal structure analysis reveals that the typical thaumatin protein exhibits a “trefoil fold,” stabilized by 8 disulfide bonds formed by 16 cysteine residues, and is a non-glycosylated protein (Ogata et al., 1992; Batalia et al., 1996; Koiwa et al., 1999). The protein structure consists of 16% α-helices, 31% β-sheets, and 53% random coils. It features a defined acidic cleft domain that includes five conserved residues: one arginine, one glutamic acid, and three aspartic acids, which likely contribute to receptor binding and the antifungal activity of TLPs. Three-dimensional structural analysis identifies three conserved domains in TLPs (Faillace et al., 2021). Domain I (residues 1–70) forms a stable β-barrel core with 11 antiparallel strands. Domain II (residues 71–140) contains an α-helix and disulfide-rich loops. Domain III (residues 140–207) comprises a β-hairpin and a coiled motif. These domains collectively form a ‘V’-shaped acidic cleft, which is crucial for ligand binding. Disulfide bonds, particularly in the C-terminus, confer remarkable stability to this structure (Faillace et al., 2021).

The V-shaped acidic cleft, formed by domains I, II, and III, possesses multiple functions. These include recognizing and binding to fungal cell wall β-1,3-glucan, thereby triggering membrane permeabilization; acting as a pattern recognition receptor to bind other pathogen-associated molecular patterns (such as lipopolysaccharides and glycoproteins); in sweet-tasting proteins, conserved acidic residues (Arg, Glu, Asp) within the cleft interact with taste bud receptors through charge-charge interactions to produce sweet taste perception; and in signal transduction, the cleft may serve as a protein-protein interaction interface, mediating the binding of TLPs with other signaling molecules.

The 16 cysteine residues form 8 disulfide bonds, contributing to the stability of the protein, making it resistant to acid/alkali denaturation and heat denaturation, and protecting it from proteolytic degradation (Smole et al., 2008). We conducted comprehensive analyses of motifs, domains, and gene structures in *Gossypium hirsutum*, *G. barbadense*, *G. raimondii*, *G. arboreum*, *Puccinia graminis*, and *Caenorhabditis elegans*, leading to the following conclusions. In *Puccinia graminis*, TLPs are often fused with glycosyl hydrolase domains, granting them polysaccharide-degrading capabilities. This allows the fungus to modulate host cell walls and maintain symbiotic balance by defending against other microorganisms. Plant TLPs with this domain exhibit the ability to degrade fungal cell walls, thereby resisting invasion, highlighting the dual functionality of TLPs. Additionally, the TLP-PA domain, present in most plants and nematodes, primarily mediates protein-protein interactions. For example, in *Arachis hypogaea* L., the interaction network of AhTLP1 (containing the TLP-PA domain) interacts with RAC13, clathrin, and FYVE domain proteins, enabling rapid responses and precise regulation to multiple environmental stresses by integrating membrane trafficking with redox regulation (Feng et al., 2024).

Structural analysis of TLPs from *Puccinia graminis* (**Figure 1F**) and *Caenorhabditis elegans* (**Figure 1G**) revealed that *Puccinia graminis* contains only 6 disulfide bonds, while both *Thaumatococcus daniellii* (**Figure 1E**) and *Caenorhabditis elegans* have 8 disulfide bonds, suggesting potential functional differences between fungal and animal TLPs. These structural features enable TLP proteins to perform diverse biological functions within the complex internal environment of plants. For instance, the allergenic TLP Mal d 2 from *Malus pumila* Mill. stabilized by eight disulfide bonds, retains its spatial conformation after heating or gastrointestinal digestion, allowing it to reach the intestine intact. There, it is taken up by antigen-presenting cells (APCs) of the immune system, triggering a systemic allergic reaction (Smole et al., 2008).

TLPs are typically small proteins. Based on molecular weight, they are classified into two types: L (Large) type TLPs, which contain 16 conserved cysteine residues and weigh 21–26 kDa, such as MtTLP1, MtTLP2, MtTLP3, MtTLP4, and MtTLP5 in *Medicago sativa* L (Saeidi and Zareie, 2020), and S (Small) type TLPs, which have peptide deletions, resulting in only 10 cysteine residues and a molecular weight of approximately 16–17 kDa (Liu et al., 2010). Analysis of the physicochemical properties of TLPs in cotton revealed a molecular weight range from 10 to 130 kDa, with a concentration between 25 and 40 kDa, conferring improved stability. The isoelectric points (pI) of cotton TLPs showed a tendency toward acidic to neutral values. Hydrophilicity was generally low, but most TLPs exhibited good hydrophilicity, providing a favorable foundation for responding to cotton stresses. Furthermore, a comparison of the physicochemical properties of TLPs from monocot *Oryza sativa* L., *Arabidopsis thaliana*, *nematodes*, and *Puccinia graminis* (**Figure 1H**) revealed that *Arabidopsis thaliana* and *Oryza sativa* L. showed similar protein length, molecular weight, and pI ranges, indicating the conserved nature of TLPs in plant systems.(See **Supplementary Table 1** for the complete dataset of physicochemical properties.) In contrast, *Puccinia graminis*, as a pathogen, exhibited specific physicochemical properties, which may be related to its adaptation for infection. Nematode TLPs were generally smaller, belonging to the S-type, which highlights the evolutionary divergence and functional adaptation of TLPs across different species. These differences in physicochemical properties offer valuable insights into the evolution of protein structure and its functional adaptation to specific ecological and pathogenic environments.

**Figure 2:**
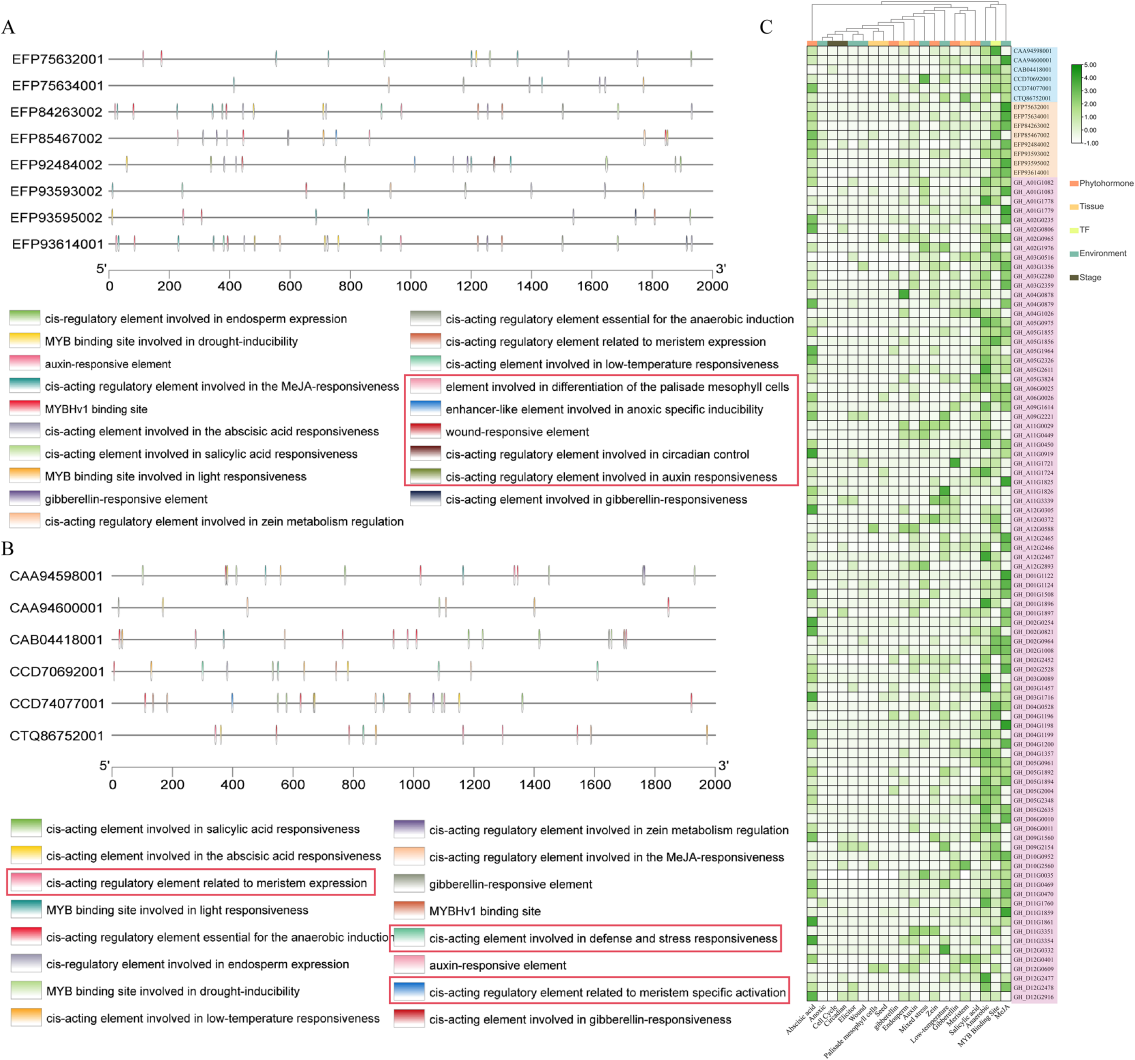
Divergence in *cis*-regulatory elements underscores functional adaptation of TLPs across kingdoms. **(A)** The upstream 2000 bp region was analyzed for cis-elements in *Puccinia graminis*. **(B)** The upstream 2000 bp region was analyzed for cis-elements in *Caenorhabditis elegans*. **(C)** Comparative analysis of cis-acting regulatory elements in *Gossypium hirsutum*, *Puccinia graminis*, and *Caenorhabditis elegans*. Red boxes highlight unique cis-acting elements. Cis-acting elements were predicted using the PlantCARE website (https://bioinformatics.psb.ugent.be/webtools/plantcare/html/).

The promoter region, as the transcriptional regulatory site, is activated by various pathogen-associated molecular patterns, abiotic stressors such as drought, waterlogging, low temperature, and heavy metals, as well as hormonal signals. It is important to note that promoter cis-element prediction is hypothesis-generating and requires experimental validation. Functional studies have demonstrated TLP responsiveness to various stimuli. For example, the *DcTLP* in *Daucus carota* is highly expressed under dehydration stress, with its promoter showing significant activation in response to drought treatment (Jung et al., 2005). Similarly, exogenous applications of jasmonic acid (JA), salicylic acid (SA), and abscisic acid (ABA) to wheat leaves led to a marked increase in *TaPR5* expression (Wang et al., 2010). Expression of AsPR5 also varied significantly over time following *Fusarium oxysporum* f. sp. *cepae* infection and treatment with exogenous stress signal molecules (Rout et al., 2016). Promoter analysis revealed that these responses are mediated by the presence of various stress-responsive cis-acting elements in TLP promoters. For instance, the 1 kb promoter region upstream of the start codon of *Allium sativum AsTLP* contains 11 phytohormones-responsive and 10 stress-responsive elements (Li et al., 2021). In bread wheat, responsive elements were classified into four categories: growth and development, light, phytohormones, and stress response elements(Sharma et al., 2022). The promoter regions of *Arabidopsis TLPs* contain five transcription factor binding sites: ASRC, CCAF, L1BX, NCS1, and WBXF. Among these, ASRC and WBXF are involved in pathogen defense, CCAF regulates the circadian clock, L1BX is a homeodomain protein recognition motif, and NCS1 represents a nodulin consensus sequence (Deihimi et al., 2012). Analysis of the upstream 2000 bp region for cis-acting elements (Lescot et al., 2002) showed that TLP transcription is regulated by multiple signals, including phytohormoness, mechanical damage, UV light, and various stress factors, such as bacterial, fungal, and viral stresses (Liu et al., 2020). Additionally, the presence of elements associated with growth and tissue-specific expression further underscores the complex and multifunctional role of TLPs, linking them to both development and immune defense. While these promoter analyses provide valuable hypotheses for TLP regulation, functional validation through techniques such as electrophoretic mobility shift assays (EMSA), chromatin immunoprecipitation (ChIP), and transgenic reporter studies is necessary to confirm the functional relevance of specific elements. Interestingly, while shared phytohormones-responsive elements are present, some elements exhibit species-specific differences (**Figure 2**). For instance, as shown in **Figure 2C**, nematodes possess elements specifically involved in controlling cell growth and division cycles, whereas plants have elements that regulate seed-specific expression, endosperm-specific regulation, flavonoid synthesis, and IAA signaling. These observations suggest that TLP functional differentiation is likely linked to species evolution and environmental adaptation.

## 4 Functions of the TLP gene family

### 4.1 Biotic stress response

#### 4.1.1 Direct antifungal action

Plants in natural environments face a range of stresses that significantly impact their growth, development, and yield, which are primarily categorized into biotic and abiotic stresses. Biotic stress arises from negative effects on plant health due to interactions with other organisms, such as pathogens, pests, weeds, and competition with other plants. Within this context, members of the *TLP* gene family play a critical role as defense proteins in plant responses to pathogen infection, engaging in complex and diverse disease resistance mechanisms. Regarding the intrinsic roles of TLPs in plant defense against fungal pathogens, research has demonstrated that TLPs can directly lyse fungal cell walls, induce hyphal tip rupture through membrane permeabilization, inhibit hyphal growth and colonization, and reduce spore viability. Their key function lies in their ability to bind and degrade glucan molecules, as well as inhibit xylanase activity, thereby providing protection against fungal diseases (Fierens et al., 2007).

As early as the late 20th century, studies on the tobacco PR5 family confirmed its direct antifungal activity, with specificity toward various fungal species. For example, the thaumatin-domain-containing *osmotin* protein effectively inhibited the growth of *Candida albicans*, *Neurospora crassa*, and *Trichoderma reesei*. Upon *Neurospora crassa* invasion, tobacco *osmotin* was induced, leading to rapid rupture of hyphal tips, mirroring the membrane-permeabilizing antifungal action of zeamatin (Vigers et al., 1992). This further corroborated the antifungal properties of *osmotin* and related proteins in *Solanum lycopersicum* L (Woloshuk et al., 1991). Subsequent studies have revealed that TLPs from a variety of plants, such as *Solanuml ycopersicum* L., *Medicago sativa* L., and *Musa acuminata* ‘(AAA)’ (including SlTLP5/6, MtTLP1-5, and BanTLP), can directly exert broad-spectrum antifungal effects by disrupting the integrity of the pathogen cell membrane, degrading cell wall components (e.g., β-1,3-glucan), or inhibiting enzymatic activity of the pathogens (Jiao et al., 2018; Saeidi and Zareie, 2020; Li et al., 2023).

The direct antifungal mechanism of TLPs is conserved in nature, with its core function lying in their capacity to target the peripheral barriers of fungal cells—the cell membrane and cell wall. Conserved domains within their structure, such as the acidic cleft, are likely responsible for recognizing and binding to fungal cell wall polysaccharides. This interaction subsequently triggers membrane permeabilization or cell wall hydrolysis, ultimately inhibiting hyphal growth and spore germination.

#### 4.1.2 Induction of systemic acquired resistance in plants

In addition to the direct antifungal actions described above, plants also employ their complex innate immune system, within which some TLPs act as elicitors to activate defense responses. Upon pathogen invasion, triggering plant defense mechanisms and activating signal transduction in immune pathways, such as phytoalexin production and activation of related defense responses (El-kereamy et al., 2011; Park and Kim, 2021).

In wheat, 50 *TLPs* showed significant differential expression upon infection with *Blumeria graminis* or *Puccinia striiformis* (Sharma et al., 2021), and *Hordeum vulgare TLPs* exhibited differential expression under *Blumeria graminis* stress (Wang et al., 2022b). PbrTLP36 protein reduced the inhibitory effect of *Diplocarpon rosae* on pollen (Zhang et al., 2024). Overexpression of *PnTLP2* from *Panax notoginseng* in tobacco significantly enhanced resistance to *Alternaria panax* (Li et al., 2020).Notably, many *TLPs* do not directly kill pathogens but instead function as elicitors to activate the plant’s endogenous immune responses. For instance, heterologous or induced expression of genes such as *ObTLP1* from *Ocimum basilicum.* (Misra et al., 2016), *ScTLP11* from *Simmondsia chinensis* (Link) *Schneider* (Zheng et al., 2024), *VuTLP* from *Vigna unguiculata* L. Walp (Jesús-Pires et al., 2024), and *RcTLP23*, *RcTLP6*, *RcTLP7* from *Rosa* sp (Su et al., 2021). has been shown to confer broad-spectrum resistance against various diseases, including *Botrytis cinerea* and *Blumeria graminis*.

TLPs also function as signaling molecules induced by ethylene, JA, and SA, and can stimulate phytohormones production, integrating into phytohormones signaling cascades (Kang et al., 2021). For example, *Zea mays* exposed to lysates of *Rhizoctonia solani* AG4-JY strains containing RsTLP3 and RsTLP10 proteins significantly upregulated *ZmPR1* expression, indicating that RsTLP3 induces expression of genes involved in SA and ethylene pathways to bolster maize resistance to diseases (Liu et al., 2024a). Overexpression of *Vitis vinifera VqTLP29* in *Arabidopsis* enhanced resistance to powdery mildew and *Pseudomonas syringae* pv. *tomato* DC3000, but decreased resistance to *Botrytis cinerea* (Yan et al., 2017). In wheat plants, TaTLP1 interacts physically with TaPR1 to regulate the burst of reactive oxygen species (ROS), thereby enhancing resistance against *Puccinia triticina (Pt)*, the causal agent of wheat leaf rust (Wang et al., 2020). Furthermore, total protein from *Populus deltoides × P. euramericana* “Nanlin895” *PeTLP* effectively inhibited *Marssonina brunnea* growth, though *in vitro* experiments with *Populus trichocarpa* did not show direct antifungal effects, suggesting PeTLP can function as a signaling molecule or elicitor in fungal resistance (Sun et al., 2020).

*TLPs* play a role in defending against fungal diseases in various species, such as *Phyllostachys edulis* (Gu et al., 2023), *Ganoderma lucidum* (Curtis) *P. Karst* (Wang et al., 2023*)*, *Fragaria ananassa* (Zhang et al., 2022), *Allium sativum* L (Anisimova et al., 2022), *Pinus radiata* D. Don (Aloi et al., 2021), *Lentinula edodes* (Ma et al., 2021), *Camellia japonica* L (Ma et al., 2021), and *Cucumis melo L.* (Liu et al., 2020). The broad-spectrum functionality of *TLPs* further solidifies their role as central hubs linking basal defense with advanced immune responses.

In summary, plant TLPs combat biotic stress through two principal strategies: direct disruption of pathogen membranes/cell walls and acting as elicitors to activate endogenous immune signaling networks. These actions frequently involve the interplay of phytohormones pathways such as salicylic acid, jasmonic acid, and ethylene, ultimately leading to the activation of conserved physicochemical defenses—including reactive oxygen species bursts, callose deposition, and lignification—to restrict pathogen spread. These shared characteristics underscore the role of TLPs as integrative hubs connecting multiple layers of the plant immune system.

#### 4.1.3 Complexity of dual functionality: TLPs in pathogens and the molecular arms race

To overcome complex plant defenses, some pathogenic organisms, including fungi, nematodes, and insects, can also produce TLPs (Mitchum et al., 2012). Secreted proteins play a pivotal role in the interaction between fungal pathogens and plants. These TLPs are secreted and function as effectors, triggering plant hypersensitivity reactions that facilitate pathogen invasion (Mitchum et al., 2012). For example, *Bursaphelenchus xylophilus* secretes TLPs to induce hypersensitivity in host trees, promoting parasitism (Kirino et al., 2020); the whitefly *Bemisia tabaci* releases the effector BtTLP into plant cells, affecting JA synthesis (Hu et al., 2025).

Comparative structural analysis reveals key differences between pathogen-derived and plant TLPs. Fungal TLPs (e.g., from *Puccinia graminis*, PDB: 7P23) typically contain only 6 disulfide bonds compared to the 8 conserved in plant TLPs, potentially conferring different stability requirements in the host-pathogen interface. Pathogen TLPs often lack the canonical V-shaped acidic cleft or exhibit modified cleft geometry that may alter ligand binding specificity. For example, in *Puccinia graminis* TLPs, key residues in the acidic cleft are substituted, weakening binding to fungal cell wall components while potentially gaining new functions in interacting with plant target proteins. Additionally, some pathogen TLPs are fused with other functional domains, for instance, *Puccinia graminis* TLPs frequently contain glycosyl hydrolase domains, enabling direct degradation of plant cell wall components. These structural adaptations reflect the evolutionary pressure on pathogens to manipulate host defenses while avoiding recognition.

Plants have evolved multiple mechanisms to recognize and counteract pathogen-derived TLPs. First, the plant immune system can detect conserved structural features of pathogen TLPs as pathogen-associated molecular patterns (PAMPs) through pattern recognition receptors, triggering PAMP-triggered immunity (PTI). For example, when *Arabidopsis* is exposed to *Rhizoctonia solani* RsTLP3 and RsTLP10 proteins, it rapidly activates MAPK cascades and upregulates *PR1* expression, mounting a defense response (Liu et al., 2024a). Second, plants may directly recognize pathogen TLPs that enter the cytoplasm through intracellular receptors, triggering effector-triggered immunity (ETI). Third, some plant TLPs may directly inhibit pathogen TLPs through competitive binding to shared targets or by forming non-functional heterocomplexes. For example, wheat TLXI can bind and inhibit fungal xylanases, directly interfering with pathogen infection (Payan et al., 2004; Li, 2012). This “effector-triggered defense” mechanism represents an important strategy for plants to counteract pathogen effectors.

The “molecular arms race” of TLPs between plants and pathogens primarily manifests in the following aspects: (1) Gene duplication and neofunctionalization: In pathogens, ancestral *TLP* genes that originally functioned in fungal cell wall remodeling undergo duplication, with some copies retaining their original functions while others neofunctionalize into effectors that manipulate host defense. For example, ten TLP genes identified in *Rhizoctonia solani* exhibit functional divergence, with some participating in fungal development and others acting as effectors that induce plant defense responses (Liu et al., 2024a); (2) Convergent evolution: Plants and pathogens have independently evolved TLPs with similar biochemical activities (such as glucanase activity) but for opposing purposes—plant TLPs for defense and pathogen TLPs for offense. This functional convergence reflects the competition between both parties at the same biochemical interface; (3) Red Queen dynamics: As plants evolve the ability to recognize pathogen TLPs, pathogens face selective pressure to diversify their TLP sequences to evade recognition while retaining essential virulence functions. This explains the remarkable sequence diversity observed in pathogen TLP repertoires—the same pathogen species often contains multiple TLP homologs with significantly divergent sequences (Clay and Kover, 1996); (4) Evolutionary trade-offs: Pathogen TLPs face conflicting selective pressures—they must retain sufficient structural similarity to maintain core biochemical functions, yet possess enough divergence to avoid plant recognition. This evolutionary trade-off shapes the sequence evolution rates of pathogen TLPs, typically manifested as the coexistence of rapidly evolving hotspots under selective pressure alongside structurally conserved regions essential for function.

The activation of host immune systems upon pathogen invasion is often leveraged in developing plant immune inducers. For instance, extracts from the endophytic fungus *Paecilomyces variotii*, such as ZNC, stimulate rutin accumulation in Solanum lycopersicum L., boosting resistance to *Botrytis cinerea* (Zhao et al., 2023), and induce the transcription of PTI genes in potatoes, enhancing resistance to late blight (Cao et al., 2021). This characteristic can be exploited by developing fungal TLPs as plant immune inducers, activating plant defenses and offering protection against pathogens.

### 4.2 Abiotic stress response

Abiotic stress, on the other hand, refers to non-biological stressors that negatively impact plant growth, including climatic factors, soil conditions, and environmental pollution. Currently, stresses such as cold, heat, drought, waterlogging, and salinity pose significant threats to crop production. *TLPs* are central to plant defense against these stresses, with their mechanisms primarily involving osmoregulation to maintain cellular homeostasis and the activation of regulatory signaling pathways.

*Ammopiptanthus nanus*, native to the harsh desert environments of Wuqi County, Xinjiang, China, and Kyrgyzstan, exhibits remarkable tolerance to environmental stress. An investigation into its genome identified 31 *TLPs*, which displayed varying transcriptional levels under low-temperature and osmotic stress conditions. Notably, overexpression of *AnTLP13* in tobacco resulted in significantly lower malondialdehyde (MDA) and relative electrolyte leakage (REL) compared to control plants. The AmWRKY14-AmTLP25 module was found to enhance winter low-temperature adaptability in *Ammopiptanthus mongolicus*, highlighting the role of TLPs in responding to cold and osmotic stress (Liu et al., 2023, 2024). *Brassica oleracea* var. *italica* BolTLP1 interacts with BolRD2, BolRD22, BolVOZ2, BolLSM1B, and BolMDH, activating plant phytohormones-mediated signaling pathways to confer tolerance to salt and drought stress in *broccoli* (He et al., 2021). Ectopic expression of *Vicia faba VfTLP4–3* and *VfTLP5* in tobacco leaves improved tolerance to stress (Zhao et al., 2024), while under heat stress, the expression of *TaTLP2-B*, *TaTLP7-D*, *TaTLP14-B1*, and *TaTLP25-B* was significantly upregulated(Sharma et al., 2022). These findings confirm that TLPs are involved in plant defense against abiotic stresses, providing valuable genetic resources for improving resilience to environmental stress.

Collectively, TLPs play significant and diverse roles in plant responses to abiotic stresses such as drought, salinity, and temperature extremes. Their mechanisms are often associated with cellular osmoregulation and the maintenance of redox homeostasis. Many TLPs are induced by abscisic acid signaling and function by interacting with other stress-responsive proteins, thereby integrating into broader stress-responsive networks to enhance overall plant tolerance. This highlights the fundamental and multifaceted regulatory functions of TLPs in abiotic stress adaptation.

### 4.3 Regulation of plant growth and development

The TLP gene family also plays a key role in plant growth and development, influencing processes such as cell division, differentiation, growth, and senescence. By regulating the expression of associated genes, TLP proteins can affect the plant’s growth rate and morphogenesis.

Transcript expression analysis of *Hordeum vulgare TLP* genes revealed differential expression patterns during various developmental stages, suggesting *TLP* involvement in tissue development. During seed germination, the transcriptional abundance of *HvTLP* genes changed significantly. For instance, *HvTLP1* and *HvTLP2* exhibited expression changes during germination, indicating a role in this process (Iqbal et al., 2020). *HvTLP8* interacts with β-glucan-dependent redox reactions, influencing barley malting quality (He et al., 2021). In *Oryza sativa* L., lines overexpressing *Rtlp2* showed a significantly higher seed setting rate and yield per plant compared to wild-type, suggesting that *Rtlp2* positively regulates grain length and weight (Du et al., 2024). Transgenic *Arabidopsis* expressing *Camellia sinensis CsTLP* exhibited increased seed yield and survival rates under drought stress (Muoki et al., 2021). Ectopic expression of *GCTLP2* in *Arabidopsis* hindered callose deposition and degradation during microsporogenesis, leading to microspore death and male sterility (Oh and Park, 2024). *RcTLP8* is specifically expressed in pistils and ovaries, suggesting a role in reproductive processes (Su et al., 2021). Additionally, *PtTLP6* is expressed specifically in the phloem (Hardtke, 2023), involved in transporting photosynthesis products, water, and nutrients. Overexpression of *PtTLP6* in *Arabidopsis* led to increased β-1,3-glucanase activity, promoting phloem cell wall thickening, accelerating phloem maturation, and resulting in earlier flowering (Guo et al., 2024).

### 4.4 Allergenicity and enzyme inhibitory activity

Some TLPs also exhibit specialized functions in certain species, such as allergenicity and enzyme inhibition.

#### 4.4.1 Allergenicity of TLPs

Both fruit allergens (Breiteneder and Radauer, 2004) and pollen allergens (Ricciardi et al., 2022) belong to various protein families, including the TLP family. The allergenic Mus a 4 from banana has been clinically and molecularly identified as a cause of allergic reactions (Suriyamoorthy et al., 2022). Eri Izumi et al. identified Pru av 2 as the first allergen from *Cerasus pseudocerasus* to trigger percutaneous sensitization in mice (Izumi et al., 2021). A 24-kDa TLP allergen, Act d 2, was detected in *green kiwifruit* (Gavrović-Jankulović et al., 2002), but not in *gold kiwifruit* (Wang et al., 2022a), indicating varietal specificity. Similarly, a 24-kDa TLP protein identified in *Vitis vinifera* also exhibits allergenic activity (Wang et al., 2022a). Other *TLP* family members confirmed as fruit allergens include Prup 2 from *peach* (Aggarwal et al., 2010), Cap a 1 from sweet pepper (*Capsicum annuum*) (Fuchs et al., 2002), and Mal d 2 from apple (Smole et al., 2008). In pollen, Jun a 3 from mountain cedar (*Juniperus ashei*) was the first allergenic TLP identified (Midoro-Horiuti et al., 2000), followed by Cup a 3, an allergenic pollen TLP isolated from *Cupressus arizonica* in 2004 (Cortegano et al., 2004).

The eight conserved disulfide bonds in the allergenic TLP Mal d 2 from apple confer exceptional stability against proteolytic degradation in the gastrointestinal tract. This structural rigidity maintains intact conformational epitopes—formed by specific amino acid sequences and three-dimensional structures—that are recognized by B cells and T cells of the immune system. The disulfide bond network folds the protein into a compact globular structure, protecting internal antigenic determinants from protease hydrolysis, allowing Mal d 2 to reach the intestinal mucosa in an immunologically intact form and trigger allergic reactions (Smole et al., 2008).

#### 4.4.2 Enzyme inhibitory activity of TLPs

Beyond their role as allergens, some TLPs exhibit remarkable enzyme inhibitory activities that have significant implications for both plant defense and pathogen virulence. These inhibitory functions reveal another dimension of TLP functionality in molecular interactions, further expanding their role as integrative hubs. Zhang Jiaying et al. identified an effector protein, Pt9029, containing a TLP domain in the wheat leaf rust pathogen *Puccinia triticina*. This protein, located in chloroplasts and possessing a chloroplast transit peptide, inhibits chloroplast ribulose-1,5-bisphosphate carboxylase/oxygenase activase (TaRCA), weakening host defense mechanisms during pathogenesis by inhibiting enzyme activity (Chang et al., 2024). During pathogenesis, this pathogen secretes the effector protein Pt9029, which contains a TLP domain. Pt9029 is translocated into plant cells and localizes to chloroplasts via its chloroplast transit peptide. Once inside chloroplasts, Pt9029 specifically inhibits ribulose-1,5-bisphosphate carboxylase/oxygenase activase (TaRCA). By inhibiting TaRCA, the pathogen weakens host defense mechanisms, as photosynthesis provides the energy and metabolic intermediates required for effective immune responses. This represents a sophisticated virulence strategy where a pathogen has evolved a TLP that mimics or competes with plant proteins to suppress defense. Conversely, plants may produce TLPs that inhibit pathogen enzymes. For example, wheat (Li, 2012) inhibits fungal xylanases, directly interfering with pathogen cell wall degradation machinery. This reciprocal enzyme inhibition exemplifies the molecular arms race between plants and pathogens, with TLPs deployed as both offensive and defensive weapons (Payan et al., 2004; Li, 2012). TLPs isolated from *Vicia faba* show inhibitory activity against human immunodeficiency virus type I (HIV-1) integrase and reverse transcriptase, with lower inhibitory effects on HIV-1 protease (Ng et al., 2002).

**Figure 3:**
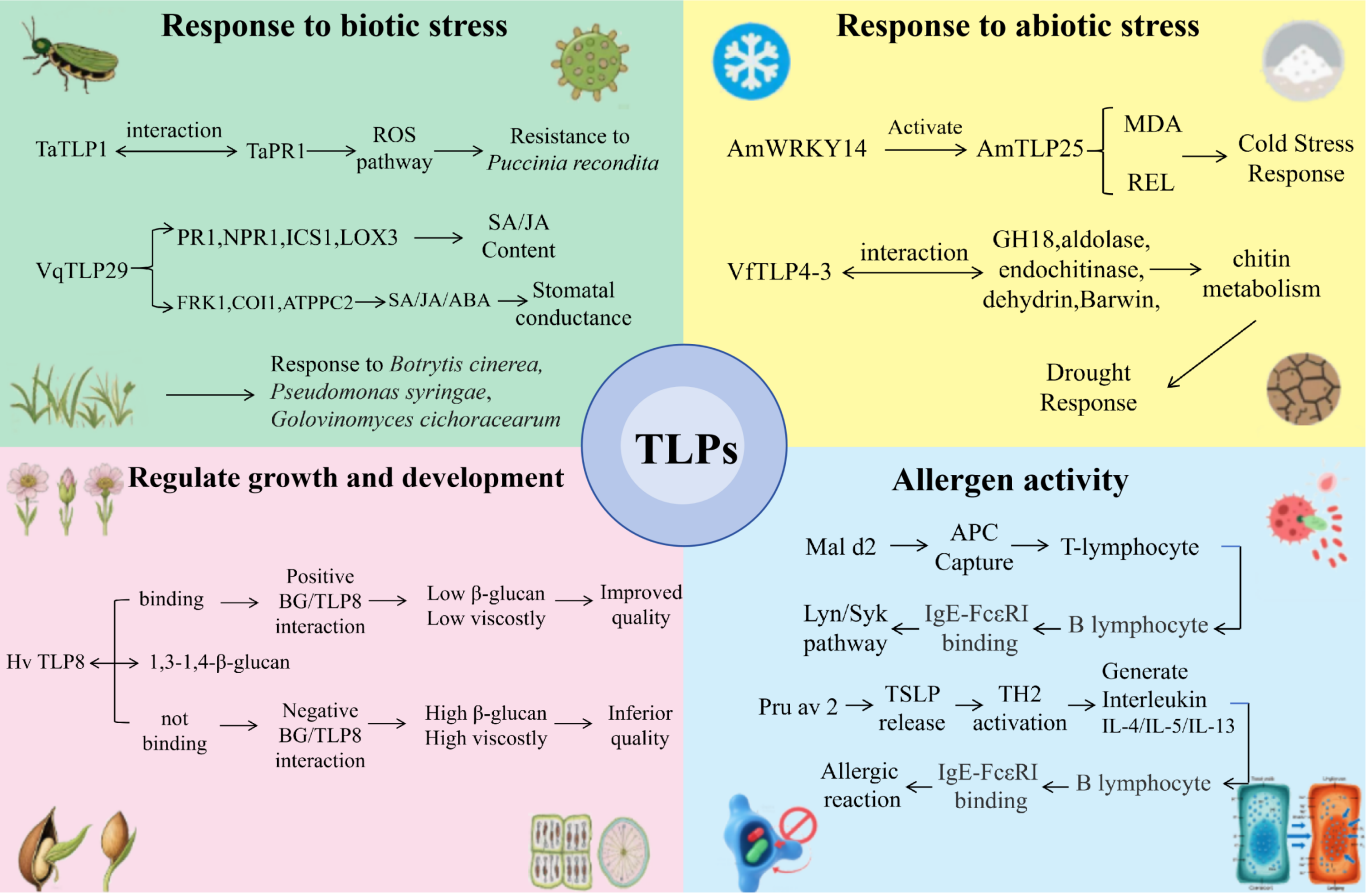
Molecular mechanisms of TLP functions.

TLP functions also exhibit species-specific variations. For instance, Mir Asif Iquebal et al. analyzed the transcriptomics of chickpea (*Cicer arietinum* L.) for imidazolinone (Imazethapyr) herbicide resistance genes and observed significant differential expression of a gene encoding thaumatin-like protein 1. This suggests that TLPs may be linked to herbicide resistance mechanisms (Iquebal et al., 2017). Similarly, Feng Lanlan et al. identified interacting proteins for *Arachis hypogaea* L. AhTLP1, revealing that AhTLP1 can physically interact with Rac-like GTP-binding protein RAC13, ribonucleoside-diphosphate reductase subunit β, clathrin heavy chain, β-adaptin-like protein, FYVE domain-containing protein, adaptor protein complex 2 subunit, and another β-adaptin-like protein. This interaction network highlights TLP involvement in diverse cellular processes (Feng et al., 2024). **Figure 3** provides a summary of the mechanisms through which plant TLPs exert their functions, while **Table 1** outlines the specific functions of various TLPs across different plant species.

**Table 1:** Functions of the TLP gene family.

Name	Sources	Function	References
osmotin	*Nicotiana tabacum* L.	Antifungal activity	(Vigers et al., 1992)
osmotin	*Solanum lycopersicum* L.	Antifungal activity	(Woloshuk et al., 1991)
*SlTLP5,SlTLP6*	*Solanum lycopersicum*	Antifungal activity	(Li et al., 2023)
*PnTLP2*	*Panax notoginseng*	Antifungal activity	(Li et al., 2020)
*PeTLP*	*Populus deltoides × P. euramericana* “Nanlin895”	Antifungal activity	(Sun et al., 2020)
*MtTLP1-MtTLP5*	*Medicago sativa* L.	Antifungal activity	(Saeidi and Zareie, 2020)
*BanTLP*	*Musa acuminata* “(AAA)”	Antifungal activity	(Jiao et al., 2018)
*PheTLPs*	*Phyllostachys edulis*	Antifungal activity	(Gu et al., 2023)
*GlTLPs*	*Ganoderma lucidum* (Curtis) *P.* Karst.	Antifungal activity	(Wang et al., 2023)
*FaTLPs*	*Fragaria ananassa*	Antifungal activity	(Zhang et al., 2022)
*AsTLPs*	*Allium sativum* L.	Antifungal activity	(Anisimova et al., 2022)
*PrTLPs*	*Pinus radiata* D. Don	Antifungal activity	(Aloi et al., 2021)
*LeTLPs*	*Lentinula edodes*	Antifungal activity	(Ma et al., 2021)
*CjTLPs*	*Camellia japonica* L.	Antifungal activity	(Ma et al., 2021)
*CmTLPs*	*Cucumis melo* L.	Antifungal activity	(Liu et al., 2020)
*AnTLPs*	*Ammopiptanthus nanus*	Low temperature and osmotic response	(Liu et al., 2023)
*AmTLP25*	*Ammopiptanthus mongolicus*	Low temperature response	(Liu et al., 2024b)
*BolTLP1*	*Brassica oleracea* L. var. *italica*	Drought response	(He et al., 2021)
*VfTLP4-3, VfTLP5*	*Vicia faba* L.	Drought response	(Zhao et al., 2024)
*TaTLP2-B,TaTLP7-D,TaTLP14-B1,TaTLP25-B*	*Triticum aestivum*	Heat stress response	(Sharma et al., 2022)
*RsTLP3,RsTLP10*	*Rhizoctonia solani*	Disease resistance response	(Liu et al., 2024a)
*ObTLP1*	*Ocimum basilicum*	Salt, dehydration, and disease resistance	(Misra et al., 2016)
*ScTLP11*	*Simmondsia chinensis* (Link) *Schneider*	Stress response	(Zheng et al., 2024)
*VuTLPs*	*Vigna unguiculata* L. Walp.	Stress response	(Jesús-Pires et al., 2024)
*RcTLP23,RcTLP6,RcTLP7*	*Rosa* sp.	Disease resistance response	(Su et al., 2021)
*TaTLPs*	*Triticum aestivum*	Disease resistance response	(Sharma et al., 2022)
*HvnTLPs*	*Hordeum vulgare* L. var. *nudum*	Disease resistance response	(Wang et al., 2022b)
*PbrTLP36*	*Pyrus bretschneideri*	Disease resistance response	(Zhang et al., 2024)
*VqTLP29*	*Vitis vinifera* L.	Disease resistance response	(Yan et al., 2017)
*HvTLPs*	*Hordeum vulgare* L.	Seed germination	(Iqbal et al., 2020)
*Rtlp2*	*Oryza sativa* L.	Growth and development, Heat stress response	(Du et al., 2024)
*CsTLP*	*Camellia sinensis* L.	Growth and development, drought response	(Muoki et al., 2021)
*GCTLP2*	*Arabidopsis thaliana (L.)Heynh.)*	Growth and development	(Oh and Park, 2024)
*RcTLP8*	*Rosa chinensis*	Growth and development	(Su et al., 2021)
Pru av 2	*Cerasus pseudocerasus*	Allergen activity	(Izumi et al., 2021)
TLP-Act d 2	*Actinidia chinensis*	Allergen activity	(Gavrović-Jankulović et al., 2002)
Prup2	*Prunus persica* L	Allergen activity	(Aggarwal et al., 2010)
Cap a 1	*Capsicum annuum*	Allergen activity	(Fuchs et al., 2002)
Mal d2	*Malus pumila Mill*	Allergen activity	(Smole et al., 2008)
Jun a 3	*Juniperus ashei*	Allergen activity	(Midoro-Horiuti et al., 2000)
Cup a 3	*Cupressus arizonica*	Allergen activity	(Cortegano et al., 2004)
*VfTLPs*	*Vicia faba* L.	Enzyme inhibitory activity	(Ng et al., 2002)
*Pt9029*	*Puccinia triticina*	Enzyme inhibitory activity	(Chang et al., 2024)
*CaTLPs*	*Cicer arietinum* L.	Herbicide resistance	(Iquebal et al., 2017)

### 4.5 Allergenicity and enzyme inhibitory activity

Throughout the preceding discussion, the common feature of TLPs functioning in multiple processes—biotic stress, abiotic stress, growth and development, and allergenicity—points to their core role as “integrative hubs.” In this section, we systematically elaborate how TLPs achieve this integrative function through three interconnected mechanisms.

#### 4.5.1 Protein-protein interaction networks

TLPs engage in physical interactions with diverse proteins through their structural domains (particularly the TLP-PA domain), forming complex signaling networks. *Arachis hypogaea* L. AhTLP1 provides a representative example: it interacts with Rac-like GTP-binding protein RAC13 (involved in reactive oxygen species signaling), ribonucleoside-diphosphate reductase subunit β (implicated in DNA synthesis during defense responses), clathrin heavy chain and β-adaptin-like proteins (involved in vesicle trafficking and receptor-mediated endocytosis), and FYVE domain-containing proteins (involved in membrane trafficking and phosphoinositide signaling) (Feng et al., 2024). This diverse interaction network places TLPs at the nexus of multiple cellular processes—signaling, membrane trafficking, and redox regulation.

In phytohormone signaling pathways, TLPs interact with key signaling node proteins. For example, *Brassica oleracea* BolTLP1 interacts with BolRD2, BolRD22, BolVOZ2, BolLSM1B, and BolMDH, proteins involved in drought response, transcriptional regulation, and metabolic processes (He et al., 2021). Through such multi-protein interaction networks, a single TLP can simultaneously influence multiple downstream pathways, achieving signal integration and coordination.

#### 4.5.2 Shared signaling nodes

TLPs can simultaneously participate in multiple phytohormone signaling pathways, functioning as shared signaling nodes. Extensive evidence demonstrates that TLP expression is regulated by multiple phytohormones including SA, JA, ET, and ABA, while TLPs themselves can feedback-regulate the synthesis and signaling of these phytohormones.

Taking *Vitis vinifera VqTLP29* as an example, its overexpression simultaneously activates SA pathway genes (*PR1, NPR1, ICS1*) and JA pathway genes (*LOX3*), while also modulating *FRK1*, *COI1*, and *ATPPC2* expression—genes involved in SA, JA, and ABA signaling pathways and stomatal regulation. This multi-pathway activation capacity enables *VqTLP29* to coordinate plant responses to different types of stress (Yan et al., 2017). Similarly, *Rhizoctonia solani* RsTLP3 can induce *ZmPR1* expression in *Zea mays*, activating SA and ETH pathways to enhance disease resistance (Kang et al., 2021; Liu et al., 2024a). This indicates that even non-plant-derived *TLPs* can integrate with plant endogenous signaling pathways to exert signal integration functions.

#### 4.5.3 Transcriptional co-regulation networks

At the transcriptional level, TLP genes form co-regulatory networks with other defense-related genes. Promoter analysis reveals that TLP gene upstream regions contain multiple stress-responsive cis-acting elements, including phytohormone-responsive elements (such as ABRE, ERE, GARE, TATC), stress-responsive elements (such as TC-rich repeats, MBS, LTR, HSE), and defense-responsive elements (such as W box, AS-1). The combination of these elements determines the responsiveness of *TLP* genes to multiple signals.

Importantly, TLP genes often exhibit co-expression patterns with defense marker genes such as *PR1*, *NPR1*, *ICS1*, and *LOX3*. For example, under pathogen infection or stress treatment, upregulation of *TLP* genes is typically accompanied by coordinated upregulation of these defense genes, indicating regulation by common transcriptional regulatory networks (Yan et al., 2017). Transcription factors from families such as WRKY, NAC, MYB, and bZIP have been confirmed to directly bind *TLP* promoters and regulate their expression. For instance, *AmWRKY14* directly regulates *AmTLP25* expression, participating in cold adaptation of *Ammopiptanthus mongolicus* (Liu et al., 2023, 2024).

Through these three mechanisms—protein-protein interaction networks, shared signaling nodes, and transcriptional co-regulation—TLPs achieve integration of multiple stress signals, coordinating plant defense and development, and solidifying their core position as central integrators of plant stress responses.

## 5 Summary and prospects

The *TLP* gene family, an ancient and multifunctional gene group in plants, plays a pivotal role in sweetness generation and various biological functions through its distinctive “TLP-fold” structure and amino acid sequence. Its intricate expression regulatory network highlights the role of *TLPs* as “integrative hubs,” integrating multiple stress signals and balancing plant defense and development. From direct antimicrobial actions to indirect regulatory functions, and from defending against environmental stress to influencing seed yield and quality, *TLP* functions are continually redefined and expanded.

Over the past five years, advanced technologies have elucidated the high-resolution structures of multiple plant TLPs, highlighting the connection between their structural features and functional roles. Concurrently, functional research has led to the screening and identification of new TLP genes across a wider range of species, enhancing their diversity and expanding their functional repertoire. Beyond their well-known role in sweetness, some plant TLPs exhibit additional biological activities, such as antipest and antioxidant properties. These findings expand the potential applications of TLPs in plant breeding, disease control, and the food industry. They not only provide a theoretical basis for developing novel sweeteners and bioactive substances but also offer insights into plant responses to environmental changes and biological defense mechanisms.

With advancements in genomics and proteomics, incorporating TLP family genes with antifungal properties into plants *via* genetic engineering can significantly boost disease resistance, providing new solutions for plant disease management. The functional attributes of TLPs also allow for improvements in plant quality traits through genetic modification. Moreover, plant TLP proteins can be utilized as carriers for drug delivery systems, targeting taste receptors to enhance drug palatability and absorption efficiency. For example, coupling drugs with TLP proteins could lead to the development of more effective oral formulations. Certain plant and fungal TLPs, along with their derived peptides, may serve as green biopesticides or efficient plant immune inducers, such as the endophytic fungal extract ZNC.

Despite these advances, several challenges and opportunities remain for future research. Further investigations should focus on exploring the structure-function relationships of plant TLPs, especially the molecular mechanisms underlying sweetness perception and other biological activities. Additionally, research should delve deeper into the metabolic pathways and regulatory networks of TLPs in plants, creating detailed, comprehensive regulatory maps. Bioinformatics tools should also be employed to integrate and analyze the growing body of TLP data, uncovering the links between functional differentiation in the TLP family and environmental adaptability. Such efforts will yield valuable research clues and targets, ultimately providing new genetic resources and theoretical foundations for plant genetic transformation and molecular breeding. 
